# In Vitro Iron Bioavailability of Brazilian Food-Based by-Products

**DOI:** 10.3390/medicines5020045

**Published:** 2018-05-16

**Authors:** Gabriela M. Chiocchetti, Elisabete A. De Nadai Fernandes, Anna A. Wawer, Susan Fairweather-Tait, Tatiana Christides

**Affiliations:** 1 Centro de Energia Nuclear na Agricultura (CENA), Universidade de São Paulo (USP), Av. Centenário, 303, 13416-000 Piracicaba, SP, Brazil; gmchiocchetti@gmail.com (G.M.C.); lis@cena.usp.br (E.A.D.N.F.); 2 Norwich Medical School, University of East Anglia, Norwich NR4 7TJ, UK; aa.wawer@gmail.com (A.A.W.); s.fairweather-tait@uea.ac.uk (S.F.-T.); 3 Department of Life and Sports Sciences, Faculty of Engineering & Science, University of Greenwich, Medway Campus, Chatham Maritime, Kent ME4 4TB, UK

**Keywords:** Caco-2 cells, iron, bioavailability, phytic acid, agro by-products, food waste, waste utilization

## Abstract

**Background**: Iron deficiency is a public health problem in many low- and middle-income countries. Introduction of agro-industrial food by-products, as additional source of nutrients, could help alleviate this micronutrient deficiency, provide alternative sources of nutrients and calories in developed countries, and be a partial solution for disposal of agro-industry by-products. **Methods**: The aim of this study was to determine iron bioavailability of 5 by-products from Brazilian agro-industry (peels from cucumber, pumpkin, and jackfruit, cupuaçu seed peel, and rice bran), using the in vitro digestion/ Caco-2 cell model; with Caco-2 cell ferritin formation as a surrogate marker of iron bioavailability. Total and dialyzable Fe, macronutrients, the concentrations of iron-uptake inhibitors (phytic acid, tannins, fiber) and their correlation with iron bioavailability were also evaluated. **Results**: The iron content of all by-products was high, but the concentration of iron and predicted bioavailability were not related. Rice bran and cupuaçu seed peel had the highest amount of phytic acid and tannins, and lowest iron bioavailability. Cucumber peels alone, and with added extrinsic Fe, and pumpkin peels with extrinsic added iron, had the highest iron bioavailability. **Conclusion**: The results suggest that cucumber and pumpkin peel could be valuable alternative sources of bioavailable Fe to reduce iron deficiency in at-risk populations.

## 1. Introduction

Iron deficiency (ID) is the most prevalent micronutrient deficiency in the world, affecting approximately 30% of the world’s population [1,2]. Although ID is a public health issue in the developed world, especially in high-risk groups such as young children and pregnant women [3], its prevalence is particularly high in developing countries. Iron deficiency anemia (IDA) affects approximately one billion people [4] and is associated with diminished work productivity and an increase in maternal and neonatal mortality [1].

In Brazil, hunger and poverty remain serious population problems, and ID and IDA are significant public health concerns. It is estimated that about 7.2 million Brazilians suffer from hunger, and 4.8 million young Brazilian children suffer from IDA [5]. The underlying cause of this problem relates more to access to food within the context of distribution and waste rather than food production [6]. Waste in the food chain is approximately 61% of total food planted, occurring in all processing phases—planting, harvesting, transportation, storage, distribution—and consumer use [7]. Fruit and vegetable peels, seeds and leaves, which are classed as by-products of food processing, and deemed inedible, are not included in the waste data. However, these by-products may be a potential source of minerals and vitamins including iron, and thus might be useful in addressing ID/IDA in Brazil. Furthermore, recent rapid growth of the world’s population has increased global demand for food sources [8]. Therefore, the transformation of food by-products to alternative food sources may be crucial not just in low and middle income countries, but worldwide.

Recently, uses for Brazilian agro-industrial food by-products have been investigated by several research centers as alternative sources of calories and micronutrients. These investigations resulted in the development of products such as biscuits, hamburgers and vegetable powders [9,10,11,12]; however, there is no data about the nutritional quality or bioavailability of the iron in these by-products. Some of them may potentially be nutritious and may contain high amounts of bioavailable iron [13,14] and thus could be considered possible sources for development of iron supplemental or fortificant products.

Iron absorption from foods is determined by a number of variables: the form of iron (i.e., heme versus non-heme iron); the modulating effect of dietary inhibitors and enhancers; and body iron status of the individual [15]. Non-heme iron is usually much less well absorbed than heme iron because its bioavailability is strongly influenced by the balance between iron inhibitors and enhancers [16]. Inhibitors include tannins, calcium, zinc, polyphenols, phytic acid and possibly fiber [15,16].

The primary aim of this study was to evaluate the non-heme iron (herein referred to as iron) bioavailability of 5 food processing by-products derived from fruits, vegetables and rice, from Brazilian agro-industrial sources, and thus their potential use as sources of supplemental iron. To achieve this, we employed the Caco-2 cell/in vitro digestion model to assess iron bioavailability, using absolute levels of cell ferritin as a surrogate marker for iron absorption, in the method developed by Glahn et al. [17]. This methodology has been validated and used in numerous studies in the last 20 years to evaluate iron bioavailability from foods and supplements [18,19,20].

The macronutrients composition, the concentration of iron absorption inhibitors (phytic acid, tannins and total dietary fiber) in the agro-industrial food-based by-products and their relation with iron bioavailability were also measured.

## 2. Materials and Methods

### 2.1. Samples

Samples of food processing by-products from fruits, vegetables and rice [cucumber peel, pumpkin peel, jackfruit peel (*Artocarpus heterophyllus*), cupuaçu seed peel (*Theobroma grandiflorum*), and rice bran] were collected directly from Brazilian agro-industries located in São Paulo and Amazonas states. The sample preparation was comprised of freeze-drying in a Thermo Savant ModulyoD lyophilizer (Thermo electron Corp., Waltham, MA, USA) and particle size reduction in a Retsch Grindomix GM200 knife mill (Retsch GmbH & Co, Haan, Germany), using polypropylene containers and a titanium knife to avoid contamination with iron.

### 2.2. Total Iron Content

The total amount of iron was determined by neutron activation analysis (NAA), recognized as a primary method of iron measurement in solid samples [21]. Analytical portions of 250 mg of food by-products were transferred to high-purity polyethylene capsules, specific for irradiation with neutrons. Ni–Cr alloy flux monitors with known composition and homogeneity [22] were intercalated with samples for monitoring neutron flux during the irradiation. Samples were irradiated for 8 h at a thermal neutron flux of 5.9 × 10^12^ cm^−2^ s^−1^, in the nuclear research reactor IEA-R1 of the Instituto de Pesquisas Energéticas e Nucleares, Comissão Nacional de Energia Nuclear (IPEN/CNEN), São Paulo. The induced radioactivity was measured after decay periods of 4, 7, 15 and 40 days, using an HPGe detector (GEM 45190, ORTEC, Aix en Provence, France) with 45% relative efficiency for the 1332 keV line of ^60^Co. Chemical elements were calculated by the k_0_-method using an in-house software package [23].

Use of certified reference materials (IAEA V-10 Hay Powder and INCT MPH-2 Mixed Polish Herbs) demonstrated a mean recovery of 101% for iron, which in within acceptable experimental standards.

### 2.3. Determination of Dialyzable Iron

The simulated digestion was according to the protocol described by Whittaker, Fox and Forbes [24]. All solutions were prepared fresh for each experiment, and all glassware used in experiments was soaked and cleaned in 10% nitric acid, followed by rinsing with 18 mΩ purity water.

The first step was gastric digestion simulation. Twenty g of each sample were homogenized in 100 mL of distilled H_2_O, the pH was adjusted to 2.0 with HCl, followed by the addition of pepsin (2 mg/g, Sigma, Saint Louis, MO, USA); these prepared samples are herein referred to as digestas. The digestas were shaken at 37 °C, 200 RPM, for 2 h. In the following step, pH was adjusted to 7.0 with KOH and twenty g of this digesta was put inside a bag made of a dialysis membrane (15,000 Dalton molecular weight, Fisher Scientific, Waltham, MA, USA). This bag was submersed in 25 mL of a solution of 0.5 M NaHCO_3_ for 30 min (shaken at 37 °C, 200 RPM). Past this time, a solution with bile (3 mg/g, Sigma, Saint Louis, MO, USA) and pancreatic digestive enzymes (0.5 mg/g, Sigma, Saint Louis, MO, USA) was added to the NaHCO_3_ solution, and the digestas were shaken at 37 °C, 200 RPM, 2 h. The amount of iron in the NaHCO_3_ solution was measured by inductively coupled plasma-optical emission spectrometry (ICP-OES).

### 2.4. Cell Culture

The TC7 Caco-2 cell clone was used for experiments between passages 39–51. This cell line has been validated for use in studies on iron metabolism and iron bioavailability [19].

Cells were grown in T75 tissue culture flasks in a humidified atmosphere with 5% of CO_2_ at 37 °C and sub-cultured every 7 days. The culture medium used was Dulbecco’s Modified Eagle Medium (DMEM) supplemented with 10% *v*/*v* fetal bovine serum, 1% penicillin–streptomycin, 4 mmol/L l-glutamine, 1% non-essential amino acids and Plasmocin 5 mg/mL and it was changed every 48 h. For experiments, cells were grown in six-well plates seeded at a density of 1 × 10^4^/cm^2^ and used 12–14 days after seeding as per the Glahn protocol [25].

Twenty-four hours prior to the initiation of in vitro digestion experiments, cell culture medium was changed to MEM without fetal bovine serum supplemented with 10 mmol/L PIPES [piperazine-*N*,*N*-bis-(2-ethanesulfonic acid)], 1% antibiotic/antimycotic solution, 11 μmol/L hydrocortisone, 0.87 μmol/L insulin, 0.02 μmol/L sodium selenite (Na_2_SeO_3_), 0.05 μmol/L triiodothyronine and 20 mg/L epidermal growth factor, in order to ensure adequate cell growth but low basal media iron levels.

### 2.5. In Vitro Digestion/Caco-2 Model to Measure Iron Bioavailability

Two experiments were carried out. In Experiment I, iron bioavailability from all the agro-industrial by-products samples was evaluated. Positive controls of Fe (25 µg) and Fe plus ascorbic acid (1:10 molar ratio) were carried out with each experiment, in addition to a “blank” control containing no food and no iron.

In Experiment II, the possible enhancing or inhibiting effects of the agro-industrial food by-products on extrinsically added iron bioavailability were investigated. For this purpose, all the agro- industrial by-products samples were analyzed with the addition of 25 µg of extrinsically added non-heme Fe (Fe solubilized in 1% HCL, High-Purity Standards, 100026-2). Controls were as in Experiment I.

The in vitro digestion followed a modified version of the protocol developed by Boato et al. [26]. All digestion solutions were prepared fresh for each experiment, and all glassware used in experiments was soaked and cleaned in 10% nitric acid, followed by rinsing with 18 mΩ purity water.

One g of each sample was added to 10 mL of pH 2 140 mmol/L NaCl and 5 mmol/L KCl followed by the addition of 0.5 mL pepsin solution (Chelex purified, concentration as previously noted), to simulate the peptic phase. The pH was readjusted to pH 2.0 with 1 mol/L HCl and the samples were shaken in a New Brunswick Orbital shaker at 37 °C, 200 RPM, for 1 h.

The intestinal digestion phase was initiated with the addition of 2.5 mL Chelex-purified bile/pancreatin solution (concentrations as previously noted) with subsequent adjustment of the pH to pH 6.9–7.0 with 1 mol/L NaHCO_3_. Following this, 1.5 mL of the above digestas were placed in a chamber suspended over a layer of Caco-2 cells grown on the bottom of the tissue culture wells of a six-well plate. The upper chamber was created using a 15,000 Dalton molecular weight cut-off dialysis membrane (Tubing Spectra/Por 7 dialysis membrane, Fisher Scientific, Waltham, MA, USA) fitted over a modified Transwell insert (Fisher Scientific, Waltham, MA, USA; the necks of the rings were shortened by 0.1 mm to remove the original filter and prevent excessive pressure on the cell monolayer) and held in place with a silicon ring (Parker 2-023S0613, Web Seal). Plates were placed on a platform-fitted multi-function 3D rotator (Fisher Scientific PSM3D, Waltham, MA, USA) set at six oscillations per minute in a 37 °C incubator with a 5% CO_2_ atmosphere at constant humidity for 60 min. Inserts were then removed, and an additional 1 mL of supplemented MEM was added to the cells, which were returned to the incubator for a further 22 h. Each food sample was tested in three separate experiments, *n* = 6 for each experiment.

At the end of each experiment, medium was removed from the wells and cells were rinsed twice with ice cold Phosphate Buffered Saline (PBS). 200 ml ice cold CelLytic (Sigma, Saint Louis, MO, USA) with 1% protease inhibitor was added to each well, and cell monolayers were removed with a cell scraper and placed in 1.8 mL Eppendorf tubes. Tubes were shaken for 15 min on a Stuart microtitre plate shaker at 1250 RPM and then spun at 6000 g for 6 min in a 5804R Eppendorf centrifuge. The supernatant was aspirated and stored at −80 °C until analysis.

The ferritin analysis was carried out using the SpectroFerritin MT Enzyme Linked Immunoassay (ELISA; RAMCO, Houston, TX, USA) on cell extraction supernatants. Absorption readings were performed at 492 nm with subtraction for background at 620 nm in a Thermo Multiscan Ascent Spectrophotometer. Protein concentration in each sample, to correct for differing cell counts per well, was measured using the Pierce Protein BCA Assay (Fisher Scientific, 23227, Waltham, MA, USA).

### 2.6. Macronutrients Composition and Analysis of Iron Absorption Inhibitors (Phytic Acid and Tannin)

Levels of macronutrients, phytic acid, tannin and total fiber were measured as follows. Phytic acid was determined according to the method described by Grynspan and Cheryan [27]. The samples were digested in 0.65 M HCl and the supernatant was eluted in an anionic resin and collected in a NaCl 0.7 M solution. Phytic acid levels were measured using the Wade’s reagent (FeCl_3_·6H_2_O and sulfoalicylic acid), and quantified by absorption readings performed at 500 nm in a spectrophotometer Femto 700 plus.

The amount of tannin was determined by the methodology described by Price, Hagerman and Butler [28], through metal extraction and colorimetric reaction with vanillin solution at 1% methanol, 8% HCl in methanol (1:1 methanol), left at 30 °C for 20 min. Absorption readings were performed at 500 nm in a spectrophotometer Femto 700 plus. The concentration of tannins was obtained from a standard catechin curve, and the results were expressed as mg/100 g catechin.

The macronutrients composition were determined by the AOAC methods.

### 2.7. Statistical Analysis

The data generated was analyzed statistically by means of one-factor analysis of variance (ANOVA), followed by Tukey’s post-hoc test to correct for multiple comparisons. Differences were considered significant at *p* < 0.05. Data analysis was performed using SigmaPlot (version 12.0, Systat Software, Inc., San Jose, CA, USA), except for data from ferritin formation, where the software Iron Data Manager (Excel version), provided by the University of Greenwich, was used.

Analysis of the relationship between ferritin formation, and inhibitors, and total iron levels, was carried out by non-linear regression and statistical significance was determined using the Delta method with *p* < 0.05 [29].

Because of ferritin level variation amongst the controls between experiments all data was normalized to the Fe Alone positive control according to the following equation:

$$y=f_{e}\times x$$

where *y* is the normalized value of x; and *x* is any given data point from experiment *e*; *f_e_* is the mean value of the positive control (Fe Alone) across all experiments divided by the mean value of the positive control (Fe Alone) for experiment e only. This method has been used in previously published studies [19,30].

## 3. Results

### 3.1. Total Amount of Fe and Dialyzable Fe

Table 1 shows the data for total iron and dialyzable iron in samples of food processing by-products.

The levels of total iron varied for the tested samples. Cupuaçu seed peel had the highest level of total iron, followed by jackfruit peel, presenting 830 and 379 µg/g of iron, respectively. Pumpkin peel, cucumber peel and rice bran contained similar amounts of iron (around 95–120 µg/g), approximately 12 and 26% of the Fe present in cupuaçu seed peel and jackfruit peel samples, respectively.

Cupuaçu seed peel also contained the highest amount of dialyzable iron (63.4 µg/g), which represented 7.64% of the total amount of iron measured. Pumpkin peel had the highest percentage of dialyzable iron: 21.4 µg/g of the iron was in the dialyzable form, representing approximately 20% of the total iron. Cucumber peel contained approximately 2.5 µg/g of dialyzable iron. Jackfruit peel and rice bran had less than 1% of total iron presented in the dialyzable form.

### 3.2. Iron Bioavailability of Agro Industrial by-Products

**Figure 1:**
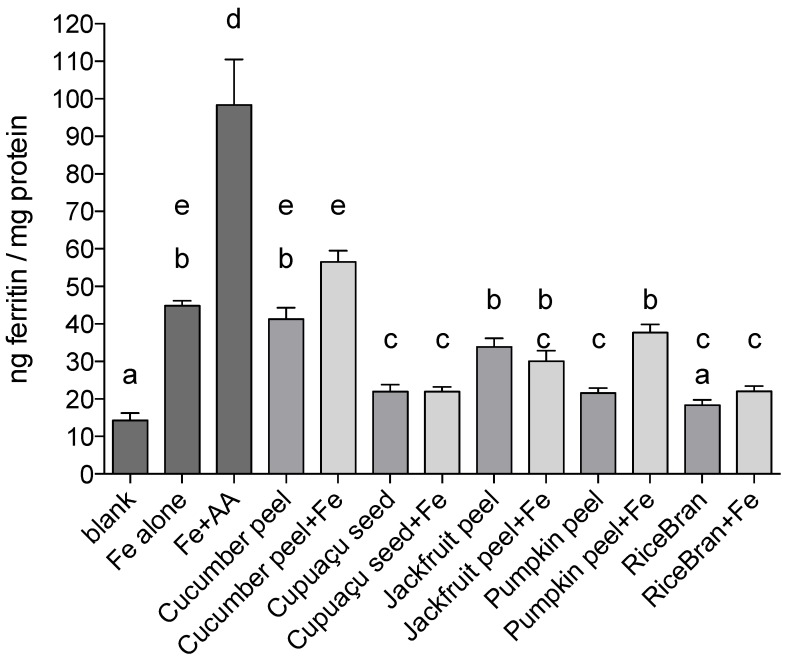
Ferritin formation (ng/mg protein) of Brazilian agro industrial by-products on a weight per weight comparative basis (one gram sample/digesta). Blank refers to digestas with no added sample, Fe alone is a positive control with the addition of inorganic Fe alone, and Fe + AA refers to iron plus ascorbic acid at a 1:10 molar ratio. All other samples are as referred to in the manuscript. Caco-2 cell ferritin formation was highest after exposure to cucumber peel + Fe. Cucumber peel, jackfruit peel, jackfruit peel + Fe and pumpkin peel + Fe treatments resulted in similar ferritin formation. In Pumpkin peel, cupuaçu seed peel, cupuaçu seed peel + Fe and rice Bran + Fe treatments, ferritin levels were similar and approximately 50% less than the response noted in the Fe alone positive control. Rice bran treated cells formed the lowest amount of ferritin: 75% less than the levels formed with Fe alone. Values are presented as means ± standard error of the mean (*n* = 18). Different letters show statistically significant differences (*p* < 0.05) between samples.

Data on iron bioavailability from the in vitro digestion/Caco-2 cell model on a weight per weight basis (one gram of each sample was used per digesta) comparative basis are presented in Figure 1.

Cucumber peel and jackfruit peel treatments resulted in the greatest uptake of iron as measured by the ferritin assay (41.3 and 33.9 ng ferritin/mg protein, respectively). Ferritin formation by Caco-2 cells treated with pumpkin peel, cupuaçu seed peel and rice bran was approximately 50% less than Fe only positive control induced ferritin formation (*p* < 0.0001 in all instances).

When Fe was added to the by-product samples, Caco-2 ferritin formation in cells treated with cucumber peel + Fe was higher when compared to the treatment with cucumber peel only, although not statistically significant (*p* = 0.0587). A stronger effect was observed in pumpkin peel samples: addition of Fe increased ferritin formation by 68% (*p* < 0.0001). The addition of Fe to Cupuaçu seed peel, jackfruit peel or rice bran samples did not affect ferritin formation significantly when compared to treatments containing by-products only; cupuaçu seed peel, jackfruit peel and rice bran, respectively.

### 3.3. Concentrations of Macronutrients and Iron Absorption Inhibitors in Agro-Industrial by-Products

Table 2 shows the data for macronutrient and energy composition, and iron absorption inhibitors, namely phytic acid, tannin and total fiber in agro-industrial food-based by-products.

The level of phytic acid varied widely between samples; rice bran had the highest amount of phytic acid (1994 mg/100 g), followed by cupuaçu seed peel (1519 mg/100 g), and the second lowest ferritin levels. Cucumber peel and pumpkin peel presented similar amounts of phytic acid (approximately 200 mg/100 g). Jackfruit peel levels of phytic acid were below the limit of detection (BLD) (<139 mg/100 g).

Rice bran and cupuaçu seed peel contained the highest amount of tannin (300 and 462 mg/100 g catechin, respectively). Levels of tannin in cucumber peel (189 mg/100 g catechin) were approximately 40% of cupuaçu seed peel samples. Jackfruit peel tannin levels were BLD (<35 mg/100 g catechin).

Cucumber peel presented the highest amount of protein and total fiber, and low concentration of lipids and carbohydrates. Pumpkin peel had the highest amount of carbohydrates, and low concentration of total fiber. Levels of dietary fiber varied between 50 and 24 g/100 g of sample; Jackfruit peel and cucumber peel had the highest amounts (50 and 46 g/100 g, respectively), followed by cupuaçu seed peel and rice bran (31 and 29 g/100 g), respectively). Pumpkin peel presented the lowest value (24 g/100 g), just 48% of the total fiber present in jackfruit peel sample.

### 3.4. Influence of Phytic Acid, Tannins and Total Iron Content on Ferritin Formation

**Figure 2:**
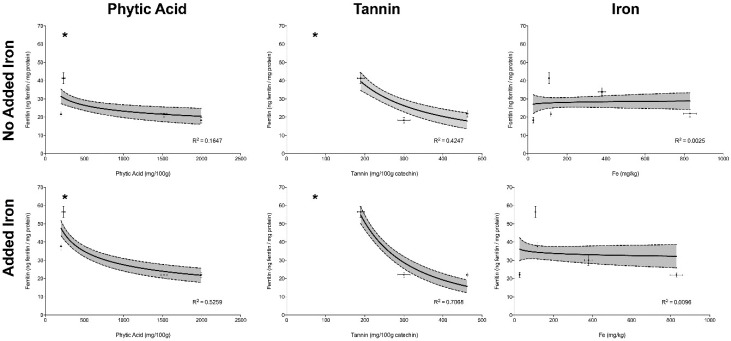
Correlation between phytic acid, tannins and total iron content versus ferritin formation in samples alone, and with added iron; R^2^ for the regression is shown for each individual inhibitor with or without added iron. Phytic acid and tannins concentrations were significantly correlated with ferritin formation in both experiments (i.e., in the absence, and presence, of added extrinsic Fe). The total iron content was not correlated with ferritin formation. Values are presented as the mean ± SEM (*n* = 18 for ferritin; *n* = 3 for phytic acid, tannin and iron). Asterisks show statistically significant differences (*p* < 0.05).

The correlation between concentrations of phytic acid, tannins and total iron versus ferritin formation by Caco-2 cells, in Experiment I (no added iron) and II (addition of 25 µg Fe) can be observed in Figure 2; R^2^ for the regression is shown for each individual inhibitor with or without added iron. Statistical significance is designated by an asterix.

Phytic acid and tannin concentrations showed an inverse correlation with ferritin formation in Experiments I, where no extrinsic iron had been added (*p* < 0.05) (i.e., the ferritin formation by Caco-2 cells was lower when phytic acid and/or tannin concentrations were higher). This effect was more pronounced when extrinsic iron was added, as in Experiment II (*p* < 0.05). The amount of total iron was not correlated with ferritin formation in either Experiment I or Experiment II (*p* > 0.05), demonstrating that high amounts of iron may not predict high iron bioavailability.

## 4. Discussion

The problem of by-product generation by agro-industries is currently of great concern, especially in a country as large as Brazil, where the agro-industry is responsible for about 6% of the Gross Domestic Product (GDP). The use of these by-products as an iron source could thus potentially reduce waste generation, contribute to iron intake in populations at risk of ID/IDA and be an alternative source of food for the growing population.

In this study, five pre-selected food-based by-products from Brazilian agro-industry were analyzed as a potential source of iron. These by-products were specifically selected because they had high iron content (≥9.7 mg/100 g), analyzed by NAA, as can be observed in Table 1. The amount of total iron in the by-product samples is similar (or higher) to plant-based foods usually recommended as sources of iron, such as green leafy vegetables (spinach: 36 mg/100 g, cauliflower: 67 mg/100 g) and beans (5 mg/100 g) [31,32]. In all these cases, it is higher than the edible part (pulp and grain), when compared with the numbers reported in the literature [33]. In similar studies, where iron concentration of several fruit peels was determined, the iron concentration was higher in the peel than in the pulp [9,34].

Although the iron content is high in these agro by-products, it is well-established that total iron levels cannot be used to predict iron bioavailability [35]. As can be observed in Table 1, the percentage of dialyzable iron is low (less than 20%), as expected for plant-based foods [36,37]. ICP-OES rather than neutron activation analysis (NAA) was used to measure iron in digestas as NAA can’t be used for liquid sample analysis, without a sample concentration step; the research literature supports the relative accuracy of these methods both in general, and in comparison with each other [38]. Iron dialyzability studies do not provide accurate information about the strength of the effect of iron inhibitors and enhancers [39]. In addition, they usually markedly underestimate iron available for absorption from large intact iron-protein complexes such as ferritin, for which there is evidence that physiological absorption does occur [40]. Indeed, ferritin may provide significant amounts of iron from plant based sources, such as the tested food by-products evaluated in this study.

In Experiment I, ferritin formation by Caco-2 cells was measured after treatment with the 5 agro food by-products on a weight per weight basis. Despite the high amount of absolute and dialyzable iron in cupuaçu seed peel when compared to pumpkin peel and rice bran, Caco-2 cells produced similar amounts of ferritin in response to cupuaçu seed peel, pumpkin peel and rice bran, corroborating that a high amount of iron does not always predict high iron absorption (Figure 2).

In Experiment II, 25 µg of Fe were added to by-product samples to assess the effects of inhibitors or enhancers in the by-products. The addition of iron to pumpkin peel increased ferritin formation by approximately 75% (*p* < 0.0001), indicating that the Fe added (or part of it) was in a soluble form and could be absorbed; this may be related to low levels of inhibitors, or the presence of enhancers such as ascorbate, or fructose that would be expected to be found in pumpkin peels. In contrast, when Fe was added to the samples of cupuaçu seed peel, jackfruit peel and rice bran, no increase in Caco-2 cell ferritin formation was observed, suggesting that there are some compounds in these samples that inhibit iron absorption.

There are several dietary factors that can interfere with iron absorption in plant-based diets. The main inhibitor of iron absorption in general diets is phytic acid. In this study, phytic acid concentrations in by-products were between 201 and 1994 mg/100 g (Table 2). These figures are consistent with findings reported by others measuring phytic acid in plant-based food, including Greiner and Konietzny [41] who found 840–1210 mg/100 g of phytic acid in oat flakes, 850–1730 mg/100 g in cooked black beans and 1270–2160 mg/100 g in cooked wild rice. The high amount of phytic acid in the rice bran (1994 mg/100 g) was expected as, in cereals, phytate is more concentrated in the bran. Others studies have found even higher levels of phytic acid in rice bran (3650 and 5800 mg/100 g) [42,43]. Consistent with the described inhibitory effects of phytates on iron bioavailability ferritin formation from rice bran was low and stayed low even when exogenous iron was added to the samples (Figure 2). Thus, rice bran-based iron supplements or fortificants would not be predicted to be good sources of additional dietary iron.

Plant foods and beverages, such as vegetables, fruits, tea, coffee and wine, are also rich in polyphenols that can act as powerful iron absorption inhibitors [26,44,45]. In cereals and legumes, polyphenols add to the inhibitory effect of phytate [16]. Tannin (a polyphenol with a high molecular weight) levels were analyzed in this study. The high concentration of both phytic acid and tannin in the cupuaçu seed peel and rice bran can explain Caco-2 cell low ferritin formation from these samples, since tannins are powerful inhibitors of iron absorption (Figure 2). Cupuaçu seed peel would therefore probably not be a good source of supplemental iron.

Interestingly, although jackfruit peel tannins levels were below the limit of detection of our assay and phytic acid levels were also low, and iron levels were moderate, iron bioavailability (as measured by ferritin) was low in comparison with other samples and positive controls (Figure 2). The high amount of fiber found in the jackfruit peel (50.4 g/100 g) (Table 2) may explain this particular result. Some studies have found a correlation between fiber content and iron binding [46,47]. However, the effect of fiber on mineral absorption is not well described [48]. Jackfruit peel and cucumber peel contained similar amounts of fiber, but when the iron was added in the Experiment II, only jackfruit peel + Fe ferritin formation was compromised, being 47% lower than cucumber peel + Fe ferritin formation (*p* = 0.0013). This contradictory result may be due to other, unmeasured, inhibitory factors present in jackfruit peel, or unmeasured enhancing factors in the cucumber peel samples.

One of the limitations of our study was that we did not measure levels of known enhancers of iron bioavailability. Established dietary enhancers of non-heme iron absorption include ascorbic acid, meat, poultry, and fish. In vitro experiments suggest fructose may also enhance non-heme iron bioavailability [30]. Levels of these substances would be predicted to vary amongst these different agro-industrial food by-products.

Additionally, iron concentrations varied between digestas (samples that had undergone in vitro digestion), thus ferritin levels reflect the weight per weight iron bioavailability of the tested products irrespective of iron content of the raw material. The findings from Experiment II, in which a known and identical amount of exogenous iron was added to all samples, are illuminating in this respect; it would be beneficial to use food processing by-products that when fortified with iron did lead to increased bioavailability, such as cucumber peel.

In conclusion, with the fast increase of the world population and the imminent decrease of natural resources, the use of by-products as an alternative source of nutrients and calories in both developing and developed countries may contribute to global food security. Some by-products, in particular cucumber and pumpkin peels, may contribute significantly to iron intake and thus potentially reduce ID/IDA in at-risk populations of Brazil and other low and middle-income countries. In addition, they might be useful as an alternative source of proteins, carbohydrates and fiber. However, the absolute amount of iron ingested does not necessarily correlate with the fraction absorbed, as indicated by our results; rather, iron bioavailability reflects the balance between iron absorption inhibitors and enhancers. We encourage the search for new by-products that can be used as alternative and inexpensive iron sources, and research in the development of new products based on cucumber and pumpkin peel. Further research, including in vivo studies to confirm our results, warrants investigation.

## Figures and Tables

**Table 1:** Total iron (µg/g) and dialyzable iron (µg/g and percentage in respect to total Fe) in samples of food processing by-products.

Samples	Total Fe (µg/g)	Dialyzable Fe	
		µg/g	% in Respect Total Fe
Cupuaçu seed peel	829.7 ± 55.4 ^a^	63.4 ± 5.12 ^a^	7.64 ± 0.36 ^b^
Jackfruit peel	379.1 ± 35.3 ^b^	2.23 ± 0.46 ^c^	0.59 ± 0.09 ^d^
Pumpkin peel	117.8 ± 5.0 ^c^	21.4 ± 0.76 ^b^	18.1 ± 0.45 ^a^
Cucumber peel	107.8 ± 2.9 ^c^	2.47 ± 0.05 ^c^	2.29 ± 0.03 ^c^
Rice bran	96.7 ± 3.8 ^c^	0.45 ± 0.02 ^d^	0.63 ± 0.15 ^d^

Values presented in dry weight (mean ± standard deviation, *n* = 3). Values within a column with unlike superscript letters are significantly different (*p* < 0.05). Total Fe jackfruit peel values have previously been published [14]; © Akadémiai Kiadó, Budapest, Hungary 2013 (with permission).

**Table 2:** Food processing by-product sample macronutrient and energy composition and inhibitor amounts (phytic acid and tannin); values presented in dry weight.

Sample	Protein	Lipids	Carbohydrates	Total Fiber	Calories	Phytic Acid	Tannin
Cucumber peel	20.4 ± 0.1 ^a^	2.34 ± 029 ^c^	8.91 ± 1.66 ^c^	46.5 ± 0.89 ^a^	138 ± 5 ^d^	233.5 ± 34.1 ^c^	189.4 ± 15.7 ^c^
Pumpkin peel	14.4 ± 0.3 ^c^	4.10 ± 0.42 ^b^	43.9 ± 1.4 ^a^	24.1 ± 0.5 ^c^	270 ± 2 ^b^	201.1 ± 22.4 ^c^	BDL *
Jackfruit peel	6.88 ± 0.04 ^d^	4.12 ± 0.42 ^b^	25.2 ± 1.2 ^b^	50.4 ± 0.9 ^a^	165 ± 9 ^c^	BDL *	BDL *
Cupuaçu seed peel	17.0 ± 0.2 ^b^	20.4 ± 1.1 ^a^	25.1 ± 4.4 ^b^	30.7 ± 1.9 ^b^	352 ± 6 ^a^	1519 ± 67 ^b^	462 ± 4.7 ^a^
Rice bran	13.0 ± 0.1 ^c^	17.6 ± 0.6 ^a^	27.5 ± 3.5 ^b^	29.4 ± 1.5 ^b^	320 ± 8 ^a^	1994 ± 24 ^a^	300 ± 27 ^b^

Values are presented as g/100 g for protein, lipids, carbohydrates and total fiber, kcal/100 g for energy, and mg/100 g for phytic acid and tannin. Values are the mean ± standard deviation, *n* = 3. Different letters show statistically significant differences (*p* < 0.05) between samples * BDL, below limit detection. (BLD for phytic acid: 139 mg/100 g; BLD for tannin: 35 mg/100 g catechin).
